# One-Year Weight Reduction With Semaglutide or Liraglutide in Clinical Practice

**DOI:** 10.1001/jamanetworkopen.2024.33326

**Published:** 2024-09-13

**Authors:** Hamlet Gasoyan, Elizabeth R. Pfoh, Rebecca Schulte, Phuc Le, W. Scott Butsch, Michael B. Rothberg

**Affiliations:** 1 Center for Value-Based Care Research, Department of Internal Medicine and Geriatrics, Primary Care Institute, Cleveland Clinic, Cleveland, Ohio; 2 Department of Medicine, Cleveland Clinic Lerner College of Medicine of Case Western Reserve University, Cleveland, Ohio; 3 Department of Quantitative Health Sciences, Lerner Research Institute, Cleveland Clinic, Cleveland, Ohio; 4 Department of Surgery, Bariatric and Metabolic Institute, Cleveland Clinic, Cleveland, Ohio; 5 Department of Internal Medicine and Geriatrics, Primary Care Institute, Cleveland Clinic, Cleveland, Ohio

## Abstract

**Importance:**

Limited data are available on long-term weight loss achieved with semaglutide or liraglutide for type 2 diabetes (T2D) or obesity in clinical practice.

**Objective:**

To document weight loss achieved with injectable forms of semaglutide or liraglutide and identify factors associated with weight reduction of 10% or greater at 1 year.

**Design, Setting, and Participants:**

This retrospective cohort study used electronic health records from a large, integrated health system in Ohio and Florida. Participants included adults with a body mass index (calculated as the weight in kilograms divided by the height in meters squared) of at least 30.0 who initiated treatment with semaglutide or liraglutide between July 1, 2015, and June 30, 2022. Follow-up was completed July 28, 2023.

**Exposure:**

Injectable forms of semaglutide or liraglutide approved for T2D or obesity.

**Main Outcomes and Measures:**

Percentage weight change and categorical weight reduction of 10% or greater at 1 year.

**Results:**

A total of 3389 patients (mean [SD] age, 50.4 [12.2] years; 1835 [54.7%] female) were identified. Of these, 1341 patients received semaglutide for T2D; 1444, liraglutide for T2D; 227, liraglutide for obesity; and 377, semaglutide for obesity. Mean (SD) percentage weight change at 1 year was −5.1% (7.8%) with semaglutide vs −2.2% (6.4%) with liraglutide (*P* < .001); −3.2% (6.8%) for T2D as a treatment indication vs −5.9% (9.0%) for obesity (*P* < .001); and −5.5% (7.5%) with persistent medication coverage (ie, a cumulative gap of less than 90 days) at 1 year vs −2.8% (7.0%) with 90 to 275 medication coverage days and −1.8% (6.7%) with fewer than 90 medication coverage days (*P* < .001). In the multivariable model, semaglutide vs liraglutide (adjusted odds ratio [AOR], 2.19 [95% CI, 1.77-2.72]), obesity as a treatment indication vs T2D (AOR, 2.46 [95% CI, 1.83-3.30]), persistent medication coverage vs 90 medication coverage days (AOR, 3.36 [95% CI, 2.52-4.54]) or 90 to 275 medication coverage days within the first year (AOR, 1.50 [95% CI, 1.10-2.06]), high dosage of the medication vs low (AOR, 1.58 [95% CI, 1.11-2.25]), and female sex (AOR, 1.57 [95% CI, 1.27-1.94]) were associated with achieving a 10% or greater weight reduction at year 1.

**Conclusions and Relevance:**

In this retrospective cohort study of 3389 patients with obesity, weight reduction at 1 year was associated with the medication’s active agent, its dosage, treatment indication, persistent medication coverage, and patient sex. Future research should focus on identifying the reasons for discontinuation of medication use and interventions aimed at improving long-term persistent coverage.

## Key Points

**Question:**

What are the observed weight outcomes and the factors associated with clinically meaningful reductions at 1 year in patients with obesity who receive injectable forms of semaglutide or liraglutide in clinical practice?

**Findings:**

In this cohort study of 3389 patients with obesity, the mean percentage of body weight change from baseline to 1 year was −5.1% for semaglutide vs −2.2% for liraglutide treatment; −3.2% for type 2 diabetes vs −5.9% for obesity indications; and −5.5% for patients with persistent medication coverage vs −2.8% with 90 to 275 coverage days and −1.8% with fewer than 90 coverage days. Factors positively associated with achieving at least 10% weight reduction at year 1 included semaglutide (vs liraglutide), obesity as a treatment indication (vs type 2 diabetes), persistent medication coverage, high dosage, and female sex.

**Meaning:**

These findings suggest that future research should focus on identifying the reasons for discontinuation of medication use and interventions aimed at improving long-term persistent coverage.

## Teaser

This cohort study examines weight outcomes at 1 year in patients with obesity who received injectable forms of liraglutide or semaglutide compared by agent, indication, dosage, and persistent coverage with the medication.

## Introduction

Obesity is a complex chronic disease that represents a significant public health and economic challenge, affecting 42% of the US adult population.^1^ It causes or exacerbates the risk of numerous major health complications, such as cardiovascular disease, type 2 diabetes (T2D), cancer, osteoarthritis, and obstructive sleep apnea.^2^ Given the medical burden of obesity and the $173 billion in associated annual costs,^3^ advances in treatment are of paramount importance.

In the past decade, 2 glucagonlike peptide-1 receptor agonists (GLP-1 RAs) were approved by the US Food and Drug Administration (FDA) for the treatment of obesity: liraglutide (2014) and semaglutide (2021).^4^ Additionally, the dual glucose-dependent insulinotropic polypeptide and GLP-1 RA agonist tirzepatide was approved by the FDA for obesity in November 2023.^5^ These medications produced clinically meaningful weight reduction in randomized clinical trials.^6,7,8^ For example, in the STEP 1 (Research Study Investigating How Well Semaglutide Works in People Suffering From Overweight or Obesity) study, once-weekly subcutaneous semaglutide, 2.4 mg, plus a lifestyle intervention produced a mean 14.9% reduction in body weight in patients with overweight or obesity (without diabetes) at 68 weeks, compared with a 2.4% weight reduction in the placebo group plus a lifestyle intervention.^6^ In the SCALE (Effect of Liraglutide on Body Weight in Non-Diabetic Obese Subjects or Overweight Subjects With Co-Morbidities) trial, once-daily subcutaneous liraglutide, 3.0 mg (plus lifestyle modification), produced a mean 8.0% weight reduction in patients with overweight or obesity (without diabetes) at 56 weeks vs a 2.6% weight reduction in the placebo group.^7^ In the STEP 8 trial, which compared once-weekly subcutaneous semaglutide, 2.4 mg, vs once-daily subcutaneous liraglutide, 3.0 mg (both groups also received counseling for diet and physical activity), over 68 weeks, adults with overweight or obesity without diabetes who received semaglutide achieved a mean body weight reduction of 15.8% vs 6.4% with liraglutide.^9^

Outside randomized clinical trial settings, data on weight loss with semaglutide or liraglutide for obesity are generally limited to 6 months of follow-up, based on brand names approved by the FDA for T2D only, or cohorts that exclude patients who did not persist with the treatment.^10,11,12,13^ Furthermore, emerging data suggest that persistent coverage with GLP-1 RAs (ie, a cumulative gap of less than 90 days)^14,15^ is difficult to achieve, but little is known about how persistence affects weight loss. Such understanding could inform the need and scope of future research, as well as clinical and policy interventions aimed at addressing the barriers to the use of these medications.^16,17^ We examined weight outcomes at 1 year in patients with obesity who received injectable forms of liraglutide or semaglutide and compared outcomes by GLP-1 RA agent, indication, dosage, and persistent coverage with the medication.

## Methods

### Study Design and Setting

Data for this retrospective cohort study were obtained from the Cleveland Clinic electronic health record (EHR) in Ohio and Florida, including linked Surescripts pharmacy dispensation records,^18^ from January 1, 2015, to July 28, 2023. The Surescripts prescription data service captures prescriptions paid for via insurance benefits, cash, coupons, or other methods, from nearly all major pharmacies and pharmacy benefit managers in the US.^19^

The study was approved by the Cleveland Clinic Institutional Review Board as minimal risk research using data collected for routine clinical practice, for which the requirement for informed consent was waived. The Strengthening the Reporting of Observational Studies in Epidemiology (STROBE) reporting guidelines were followed.

### Study Participants

**Figure 1.**
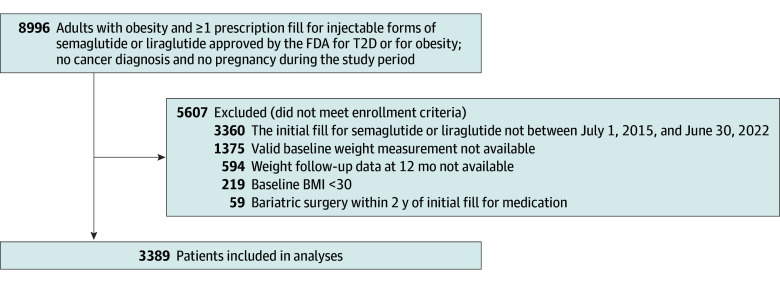
Identification of Eligible Patients for Inclusion BMI indicates body mass index (calculated as the weight in kilograms divided by the height in meters squared); FDA, US Food and Drug Administration; and T2D, type 2 diabetes.

We identified adult patients (aged ≥18 years) who filled an initial prescription for injectable semaglutide or liraglutide from July 1, 2015, to June 30, 2022, and had a body mass index (BMI; calculated as the weight in kilograms divided by the height in meters squared) of at least 30.0, recorded on the date of treatment initiation (index date) or during the latest available primary care visit before the index date. To ensure that these were initial fills, we excluded patients prescribed these medications between January 1 and June 30, 2015. Individuals were required to have a follow-up weight measurement at least 12 months after the treatment initiation to be included in the study. Patients who were pregnant, had cancer diagnoses during the study period, or underwent bariatric surgery within 2 years of the initial medication fill were excluded (Figure 1). This study captured fills for injectable forms of semaglutide and liraglutide under the brand names approved by the FDA for obesity (semaglutide, 1.7 or 2.4 mg [Wegovy], and liraglutide, 3.0 mg [Saxenda]), as well as those approved for T2D (semaglutide, 0.5, 1.0, or 2.0 mg [Ozempic], and liraglutide, 1.2 or 1.8 mg [Victoza]), including all starting doses.

### Study Variables

The primary outcome measures in this study were (1) percentage of weight change at 1 year following initiation of injectable semaglutide or liraglutide treatment and (2) categorical weight loss of 10% or greater at 1 year, given that sustained weight loss of 10% or more in patients with obesity has a major beneficial impact on obesity-related comorbidities.^20,21^ Baseline BMI was calculated using the weight data captured on the index date or, if not available, within 6 months before the index date. Body weight at 1 year was determined by the first weight measurement captured between 12 and 18 months. If no weight measurement was available after 12 months, the last weight measurement less than 3 months before the 1-year follow-up time was carried forward; otherwise, the 1-year weight measurement was considered missing. We also captured available weight data from baseline through 18 months (in 1-month increments) to compare the weight trajectories by GLP-1 RA agent. The end of the follow-up period for outcome ascertainment was July 28, 2023.

Medications were classified by their active agent (ie, semaglutide vs liraglutide) and indication (ie, for obesity vs T2D). For individuals who switched between these medications (83 for different brand name or agent in the first vs last fill within the first year), the brand name and active agent were classified based on the medication with the largest number of covered days within the first year. Medication indication was categorized based on the brand name and presence of T2D. Specifically, if a patient received semaglutide or liraglutide with a brand name approved for obesity or a brand name approved for T2D but without a documented diagnosis of T2D (indicating off-label use for obesity),^22^ the indication was captured as for obesity; otherwise, the indication was for T2D. The presence of T2D was defined by either a glycated hemoglobin level of at least 6.5% (to convert to proportion of total hemoglobin, multiply by 0.01) or the presence of a diagnostic code for T2D (*International Classification of Diseases, Ninth Revision*, code 250.X0 or 250.X2 or *International Statistical Classification of Diseases, Tenth Revision,* code E11.X). We also created a dichotomous variable for medication maintenance dosage with semaglutide, 1.7, 2.0, or 2.4 mg, and liraglutide, 3.0 mg, classified as high and all other dosages classified as low. The maintenance dosage was determined by the medication fills with the largest number of covered days within the first year. Previous receipt of other antiobesity medication approved by the FDA for chronic weight management (including phentermine-topiramate, naltrexone-bupropion, and orlistat) before the initial fill for liraglutide or semaglutide (index date) was also captured via binary (yes or no) variable.

Persistent coverage with injectable semaglutide and liraglutide use at 1 year was defined as a cumulative gap of less than 90 days within the first year after the initial prescription fill.^14,23^ Patients who switched between injectable forms of liraglutide and semaglutide but had a cumulative gap of less than 90 days were considered to have persistent coverage at 1 year. We grouped patients with nonpersistent coverage into those who had 90 to 275 medication coverage days within the first year and those who had fewer than 90 medication coverage days.

Sociodemographic variables, including patients’ age, sex, race and ethnicity, primary payer type, and Area Deprivation Index (ADI) based on census block group neighborhood-level data^24^ were recorded from the primary care visit closest to the index date. Data on sex and race and ethnicity were based on patient self-report using fixed categories. Race and ethnicity were categorized into Black, Hispanic, White, and other groups (including American Indian or Alaska Native, Asian, Native Hawaiian or Other Pacific Islander, multiracial, and other); these data were included because they could be associated with both exposure and study outcomes. Area Deprivation Index percentiles were based on a nationwide ranking from 1 to 100, where an ADI with a ranking of 1 indicates the lowest level of disadvantage.^24^ Area Deprivation Index rankings were grouped into quartiles. Payer type was categorized as private, Medicare, Medicaid, self-pay, and other. Age-adjusted Charlson Comorbidity Index was also captured from the EHR.^25^

### Statistical Analysis

Means and SDs were used to summarize normally distributed data, and medians and IQRs were used for data that were not normally distributed. Pearson χ^2^ test, 2-sample *t* test, and Wilcoxon rank sum test were used for comparisons of baseline variables.^26^ The mean percentage weight change at 1 year was compared using a 2-sample *t* test or analysis of variance.^26^

A multivariable logistic regression model was used to examine the association of 10% or greater weight loss at 1 year with the following independent variables: GLP-1 RA agent, medication indication, dosage, persistent coverage with GLP-1 RA treatment at 1 year, age, sex, race and ethnicity, primary payer type, ADI quartile, baseline BMI, age-adjusted Charlson Comorbidity Index, and previous use of other antiobesity medications. In the multivariable model, individuals with unknown race and ethnicity, primary payer type, or ADI quartile were included as a separate category. All statistical testing was 2 tailed with α = .05 used to determine statistical significance. All analyses were conducted in R, version 4.2.1, (R Program for Statistical Computing).

To assess the robustness of the identified association between medication and 10% or greater weight loss at 1 year, we conducted sensitivity analysis. For these, we excluded the patients who switched between the medications of interest from the multivariable logistic regression model.

## Results

This study included 3389 patients who filled an initial prescription for injectable semaglutide (n = 1718) or liraglutide (n = 1671) from July 1, 2015, to June 30, 2022. Mean (SD) age was 50.4 (12.2) years, and median baseline BMI was 38.5 (IQR, 34.4-44.3). A total of 1853 patients (54.7%) were female and 1536 (45.3%) were male, and 2785 (82.2%) had T2D as a treatment indication. In terms of race and ethnicity, 689 patients (20.3%) were Black, 237 (7.0%) were Hispanic, 2320 (68.5%) were White, and 116 (3.4%) were of other race or ethnicity. Most patients were privately insured (2202 [65.0%]), 631 (18.6%) had Medicare, and 468 (13.8%) had Medicaid as a primary payer; and 958 (28.3%) resided in an area in the most disadvantaged quartile of the ADI (Table 1).

**Table 1.** Characteristics of Patients Who Initiated Treatment With Injectable Semaglutide or Liraglutide for T2D or Obesity

Characteristic	Treatment group, No. (%)^a^			*P* value^b^
	Overall (N = 3389)	Liraglutide (n = 1671)	Semaglutide (n = 1718)	
Age, mean (SD), y	50.4 (12.2)	50.7 (12.2)	50.1 (12.3)	.16
Sex				
Female	1853 (54.7)	928 (55.5)	925 (53.8)	.32
Male	1536 (45.3)	743 (44.5)	793 (46.2)	
Race and ethnicity				
Black	689 (20.3)	397 (23.8)	292 (17.0)	<.001
Hispanic	237 (7.0)	115 (6.9)	122 (7.1)	
White	2320 (68.5)	1090 (65.2)	1230 (71.6)	
Other^c^	116 (3.4)	54 (3.2)	62 (3.6)	
Not reported	27 (0.8)	15 (0.9)	12 (0.7)	
Primary payer				
Private	2202 (65.0)	939 (56.2)	1263 (73.5)	<.001
Medicare	631 (18.6)	321 (19.2)	310 (18.0)	
Medicaid	468 (13.8)	357 (21.4)	111 (6.5)	
Self-pay	50 (1.5)	30 (1.8)	20 (1.2)	
Other	30 (0.9)	21 (1.3)	9 (0.5)	
Unknown	8 (0.2)	3 (0.2)	5 (0.3)	
ADI quartile^d^				
1-25	393 (11.6)	157 (9.4)	236 (13.7)	<.001
26-50	832 (24.6)	370 (22.1)	462 (26.9)	
51-75	1011 (29.8)	496 (29.7)	515 (30.0)	
76-100	958 (28.3)	565 (33.8)	393 (22.9)	
Unknown	195 (5.8)	83 (5.0)	112 (6.5)	
Charlson Comorbidity Index, median (IQR)	3 (2-4)	3 (2-4)	3 (1-4)	<.001
Baseline BMI, median (IQR)	38.5 (34.4-44.3)	38.6 (34.7-44.9)	38.5 (34.2-43.6)	.03
Indication^e^				
T2D	2785 (82.2)	1444 (86.4)	1341 (78.1)	<.001
Obesity	604 (17.8)	227 (13.6)	377 (21.9)	
Persistent coverage at 1 y^f^				
Persistent coverage	1381 (40.7)	595 (35.6)	786 (45.8)	<.001
90-275 d Medication coverage	1254 (37.0)	708 (42.4)	546 (31.8)	
<90 d Medication coverage	754 (22.2)	368 (22.0)	386 (22.5)	
Received other AOM before index date^g^	195 (5.8)	81 (4.8)	114 (6.6)	.03

Abbreviations: ADI, Area Deprivation Index; AOM, antiobesity medication; BMI, body mass index (calculated as the weight in kilograms divided by the height in meters squared); T2D, type 2 diabetes.

^a^ Percentages have been rounded and may not total 100.

^b^ Based on Pearson χ^2^ test, 2-sample *t* test, or Wilcoxon rank sum test.

^c^ Includes American Indian or Alaska Native, Asian, Native Hawaiian or Other Pacific Islander, multiracial, and other.

^d^ Quartiles were structured by ranking the ADI from low to high nationally, where an ADI with a ranking of 1 indicates the lowest level of disadvantage and 100, the highest.

^e^ Categorized based on the brand name and presence of T2D.

^f^ Defined as a cumulative gap of fewer than 90 days within the first year after the initial prescription fill. Nonpersistent coverage was grouped into 90 to 275 medication coverage days within the first year and fewer than 90 coverage days.

^g^ Includes receipt of phentermine-topiramate, naltrexone-bupropion, or orlistat before the initial fill for liraglutide or semaglutide.

Overall, 1341 patients (39.6%) received semaglutide indicated for T2D, 1444 (42.6%) received liraglutide for T2D, 227 (6.7%) received liraglutide for obesity, and 377 (11.1%) received semaglutide for obesity. Four in 10 patients (1381 [40.7%]) in the overall cohort had persistent medication coverage at 1 year, including 786 (45.8%) receiving semaglutide vs 595 (35.6%) receiving liraglutide (*P* < .001) (Table 1).

**Figure 2.**
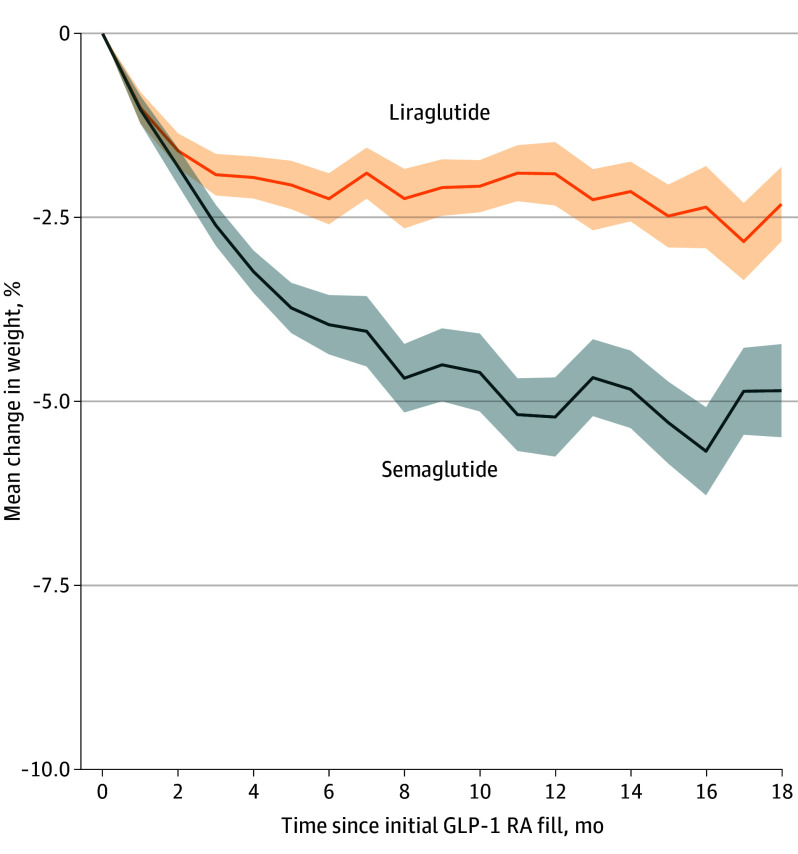
Mean Percentage Weight Reduction by Agent From Initiation of Medication Use During Study Follow-Up Available weight measurements were captured from baseline through 18 months in 1-month increments. Shaded areas indicate 95% CIs. GLP-1 RA indicates glucagonlike peptide-1 receptor agonists.

Mean (SD) percentage weight change at 1 year was −3.7% (7.3%) in the study cohort; it was −5.1% (7.8%) with semaglutide vs −2.2% (6.4%) with liraglutide (*P* < .001); −3.2% (6.8%) in patients who received the medication for T2D vs −5.9% (9.0%) for obesity (*P* < .001); −5.5% (7.5%) in patients with persistent medication coverage at 1 year vs −2.8% (7.0%) with 90 to 275 medication coverage days within the first year and −1.8% (6.7%) with fewer than 90 medication coverage days (*P* < .001); and −3.5% (7.1%) with a low maintenance dosage vs −6.6% (9.1%) with a high dosage (*P* < .001). Figure 2 presents the mean percentage weight reduction by GLP-1 RA agent from initiation of the medication use during follow-up.

Mean percentage body weight change from baseline to 1 year by medication, indication, and persistence of coverage at 1 year is presented in eTable 1 in Supplement 1. Notably, among patients with persistent coverage with their medication at year 1 (n = 1381), the mean (SD) percentage reduction in body weight was −12.9% (8.3%) with semaglutide for obesity, −5.9% (7.3%) with semaglutide for T2D, −5.6% (6.9%) with liraglutide for obesity, and −3.1% (6.1%) with liraglutide for T2D.

**Figure 3.**
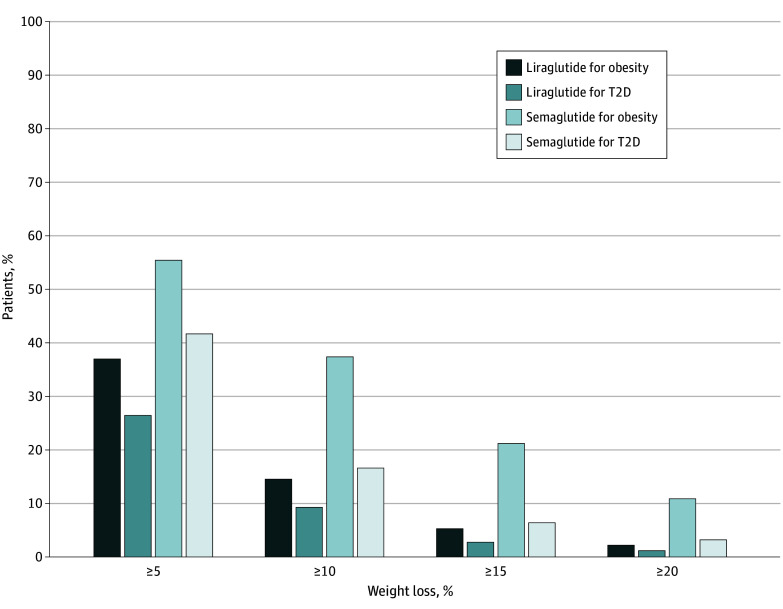
Cumulative Distribution of Categorical Weight Reduction Percentage at 1 Year by Agent and Indication in the Overall Cohort Medication indication was categorized based on the brand name and presence of type 2 diabetes (T2D).

Figure 3 and eTable 2 in Supplement 1 present the cumulative distribution of categorical percentage of weight reduction in the overall cohort by GLP-1 RA medication and indication. Overall, 141 patients (37.4%) receiving semaglutide for obesity achieved 10% or greater body weight reduction vs 223 (16.6%) of those receiving semaglutide for T2D, 33 (14.5%) of those receiving liraglutide for obesity, and 134 (9.3%) of those receiving liraglutide for T2D. Among patients with persistent medication coverage at year 1, the proportion who achieved at least 10% weight reduction was 86 of 141 (61.0%) receiving semaglutide for obesity, 149 of 645 (23.1%) receiving semaglutide for T2D, 12 of 42 (28.6%) receiving liraglutide for obesity, and 68 of 553 (12.3%) with liraglutide for T2D (eFigure in Supplement 1).

In the multivariable model, semaglutide vs liraglutide (adjusted odds ratio [AOR], 2.19 [95% CI, 1.77-2.72]), obesity as a treatment indication vs T2D (AOR, 2.46, 95% CI, 1.83-3.30), persistent coverage for 1 year vs fewer than 90 medication coverage days (AOR, 3.36 [95% CI, 2.52-4.54]) or 90 to 275 medication coverage days (AOR, 1.50 [95% CI, 1.10-2.06]), high vs low dosage of medication (AOR, 1.58 [95% CI, 1.11-2.25]), and female sex (AOR, 1.57 [95% CI, 1.27-1.94]) were positively associated with achieving 10% or greater weight reduction at year 1. A 1-unit increase in baseline BMI was associated with 2% higher odds (AOR, 1.02 [95% CI, 1.01-1.03]) of achieving 10% or greater weight reduction at year 1 (Table 2). These findings were supported by the sensitivity analysis, after excluding 83 patients who switched between the medications of interest from the multivariable model (eTable 3 in Supplement 1).

**Table 2.** Association of GLP-1 RA Agent, Indication, Dosage, and Persistent Coverage at 1 Year With ≥10% Weight Loss at 1 Year (N = 3389)

Characteristic	AOR (95% CI)^a^	*P* value
GLP-1 RA agent		
Liraglutide	1 [Reference]	<.001
Semaglutide	2.19 (1.77-2.72)	
Indication^b^		
T2D	1 [Reference]	<.001
Obesity	2.46 (1.83-3.30)	
Medication dosage^c^		
Low	1 [Reference]	.01
High	1.58 (1.11-2.25)	
Baseline BMI (1-unit increase)	1.02 (1.01-1.03)	.005
Medication coverage at 1 y^d^		
<90 d	1 [Reference]	<.001
90-275 d	1.50 (1.10-2.06)	
Persistent	3.36 (2.52-4.54)	
Age (1-y increase)	1.01 (1.00-1.02)	.26
Sex		
Female	1.57 (1.27-1.94)	<.001
Male	1 [Reference]	
Race/ethnicity		
Black	0.73 (0.55-0.97)	.14
Hispanic	1.00 (0.66-1.47)	
White	1 [Reference]	
Other^e^	1.27 (0.75-2.07)	
Not reported	1.56 (0.50-4.00)	
Charlson Comorbidity Index (1-unit increase)	0.98 (0.92-1.05)	.61
ADI quartile^f^		
1-25	1 [Reference]	.38
26-50	1.24 (0.89-1.74)	
51-75	0.98 (0.70-1.38)	
76-100	1.14 (0.80-1.64)	
Unknown	0.95 (0.57-1.55)	
Primary payer		
Private	1 [Reference]	.26
Medicare	1.36 (1.01-1.83)	
Medicaid	1.05 (0.74-1.47)	
Self-pay	0.44 (0.10-1.24)	
Other	1.22 (0.35-3.30)	
Unknown	0.94 (0.05-5.91)	
Receipt of other AOM before index date^g^		
None	1 [Reference]	.90
Prior use	0.98 (0.66-1.43)	

Abbreviations: ADI, Area Deprivation Index; AOM, anti-obesity medication; AOR, adjusted odds ratio; BMI, body mass index (calculated as the weight in kilograms divided by the height in meters squared); GLP-1 RA, glucagonlike peptide-1 receptor agonist; T2D, type 2 diabetes.

^a^ Based on a multivariable logistic regression model with the following independent predictors: GLP-1 RA agent, medication indication, dosage, persistent coverage with GLP-1 RAs at 1 year, age, sex, race and ethnicity, primary payer type, ADI quartile, baseline BMI, age-adjusted Charlson Comorbidity Index, and previous use of other antiobesity medication.

^b^ Categorized based on the brand name and presence of T2D.

^c^ Dichotomized with semaglutide, 1.7, 2.0, or 2.4 mg, and liraglutide, 3.0 mg, classified as high and all other dosages classified as low.

^d^ Defined as a cumulative gap of less than 90 days within the first year after the initial prescription fill. Nonpersistent coverage was grouped into 90 to 275 medication coverage days within the first year and fewer than 90 coverage days.

^e^ Includes American Indian or Alaska Native, Asian, Native Hawaiian or Other Pacific Islander, multiracial, and other.

^f^ Quartiles were structured by ranking the ADI from low to high nationally, where an ADI with a ranking of 1 indicates the lowest level of disadvantage and 100, the highest.

^g^ Includes receipt of phentermine-topiramate, naltrexone-bupropion, or orlistat before the initial fill for liraglutide or semaglutide.

## Discussion

In this large cohort of patients with obesity who initiated treatment with injectable semaglutide or liraglutide, patients lost an average of 3.7% body weight at 1 year. Weight loss varied significantly by the medication, indication, dosage, and persistence of medication coverage. In the multivariable analysis, patients who received semaglutide (vs liraglutide), had a high dosage of the medication (vs low), had obesity (vs T2D) as treatment indication, had persistent medication coverage or 90 to 275 medication coverage days within the first year (vs <90 medication coverage days), had higher baseline BMI, and were female (vs male) had significantly higher odds of achieving 10% or greater weight reduction at 1 year.

Recently, injectable forms of semaglutide have been more commonly prescribed, in part related to their ability to demonstrate clinically meaningful weight outcomes. As a result of its superiority, the demand for the medication has skyrocketed, leading to notable drug shortages.^22,27^ Patients have high expectations to achieve substantial weight reductions with GLP-1 RA medications.^27^ Our clinical findings suggest that this was not the case for most patients in our cohort; however, those who persisted with the medication coverage experienced weight losses comparable with those shown in corresponding clinical trials.^6,9,28^ Unfortunately, only 4 in 10 patients in this cohort had persistent medication coverage at 1 year, which underlines the current challenges with treating T2D and obesity with these highly effective medications. High out-of-pocket costs, insurance coverage–related issues, medication supply shortages, adverse effects, and weight reduction not meeting the patient’s expectations could be the reasons for this, warranting future studies on determinants of nonpersistent coverage with these medications.^14,15^ However, 61.0% of the patients who received semaglutide for obesity and with persistent coverage at 1 year achieved 10% or greater weight loss, which is only slightly less than the proportion in the STEP 1 study, where 69.1% of participants achieved at least 10% weight reduction at 68 weeks.^6^

Patients who received their medication for T2D in this study had significantly lower odds of achieving 10% or greater weight reduction at year 1, compared with those who received it for obesity, after controlling for relevant sociodemographic and clinical factors, including the GLP-1 RA agent, medication dosage, persistent coverage at 1 year, and baseline BMI. The smaller weight reduction for treatment of T2D is consistent with the results of STEP 1 and STEP 2 studies in which the placebo-adjusted mean weight reduction in patients with overweight or obesity with semaglutide, 2.4 mg, was 12.5% in those without T2D (STEP 1) vs 6.2% with T2D (STEP 2) at 68 weeks.^6,28^ Potential explanations include background diabetes medications that could promote weight gain, hyperinsulinemia, altered microbiota or genetic predisposition, and a decrease in glycosuria in patients with T2D.^29^

Furthermore, receipt of a higher dose of semaglutide or liraglutide and female sex were positively associated with a 10% or greater weight reduction at year 1. This is consistent with subgroup analyses of STEP clinical trials,^30^ as well as observational data among individuals prescribed semaglutide dosages of 0.25 to 2.0 mg/wk,^31^ but the mechanisms underlying this discrepancy are unknown. Our findings provide timely data on longer-term weight outcomes in patients receiving treatment with injectable semaglutide or liraglutide for obesity or T2D and identify key characteristics that could inform the probability of achieving sustained weight loss of a magnitude large enough to provide clinically significant health benefits.^20,21^

### Strengths and Limitations

Strengths of this study include its large and diverse sample (including 20.3% Black and 7.0% Hispanic individuals) comprising multiple years of data, integration of prescription dispensation data from Surescripts with clinical information,^19,32^ and inclusion of all patients who received the medication, whether or not they achieved persistent coverage. This study also has some limitations. The present study used data from EHRs, including Surescripts dispensed prescription data, and included adult patients in Ohio and Florida in a single large integrated health system. Patient characteristics and health care delivery patterns may vary across the US, which may limit the generalizability of our findings. During the study period, shortages of semaglutide were reported, which could have limited the ability of patients to continuously access the medication. Some of the recorded weight loss achieved may be associated with other interventions (eg, lifestyle interventions and nutritional counseling); nevertheless, the studied medications have been approved to be used in addition to diet and exercise. This study did not capture concomitant medications that are known to affect body weight, such as insulin, or attendance to lifestyle interventions and nutritional counseling. Our database did not capture patient- and clinician-related factors (such as discontinuation of the medication therapy due to inadequate blood glucose level control, weight reduction, adverse effects, or coverage issues),^16,33^ and these could not be examined in this study. Furthermore, using the EHR data might have increased susceptibility to coding errors.

## Conclusions

In this retrospective cohort study of 3389 patients with obesity treated with injectable forms of semaglutide or liraglutide in a regular clinical setting, achieving sustained weight reduction of 10% or more was associated with the GLP-1 RA agent, treatment indication, medication dosage, sex, and persistence with medication coverage. Future research should focus on identifying the reasons for discontinuation of medication use and interventions aimed at improving long-term persistent coverage.

## Notes

Supplement 1. **eTable 1.** Mean Percent Body Weight Change at 1 Year by GLP-1 RA Agent, Indication, and Persistence at 1 Year **eTable 2.** Cumulative Distribution of Categorical Weight Loss at 1 Year in the Overall Cohort **eTable 3.** Results of the Sensitivity Analysis, After Excluding Patients Who Switched Between the Medications of Interest From the Multivariable Model **eFigure.** Cumulative Distribution of Categorical Weight Reduction Percentage at 1 Year Among Patients Who Persisted With the Medications of Interest

Supplement 2.
Data Sharing Statement
