# Supplementary material for: One-Year Weight Reduction With Semaglutide or Liraglutide in Clinical Practice
Source: JAMA Netw Open. 2024 Sep 13;7(9):e2433326. doi: 10.1001/jamanetworkopen.2024.33326 (PMC11400221; doi:10.1001/jamanetworkopen.2024.33326)
Supplement: Supplement 1. — eTable 1. Mean Percent Body Weight Change at 1 Year by GLP-1 RA Agent, Indication, and Persistence at 1 Year eTable 2. Cumulative Distribution of Categorical Weight Loss at 1 Year in the Overall Cohort eTable 3. Results of the Sensitivity Analysis, After Excluding Patients Who Switched Between the Medications of Interest From the Multivariable Model eFigure. Cumulative Distribution of Categorical Weight Reduction Percentage at 1 Year Among Patients Who Persisted With the Medications of Interest [file jamanetwopen-e2433326-s001.pdf]

Supplementary Online Content

Gasoyan H, Pfoh ER, Schulte R, Le P, Butsch WS, Rothberg MB. One-year weight reduction with semaglutide or liraglutide in clinical practice. *JAMA Netw Open*. 2024;7(9):e2433326. doi:10.1001/jamanetworkopen.2024.33326

eTable 1. Mean Percent Body Weight Change at 1 Year by GLP-1 RA Agent, Indication, and Persistence at 1 Year

eTable 2. Cumulative Distribution of Categorical Weight Loss at 1 Year in the Overall Cohort

eTable 3. Results of the Sensitivity Analysis, After Excluding Patients Who Switched Between the Medications of Interest From the Multivariable Model

eFigure. Cumulative Distribution of Categorical Weight Reduction Percentage at 1 Year Among Patients Who Persisted With the Medications of Interest

This supplementary material has been provided by the authors to give readers additional information about their work.

© 2024 Gasoyan H, et al. *JAMA Network Open*.

eTable 1. Mean Percent Body Weight Change at 1 Year by GLP-1 RA Agent, Indication, and Persistence at 1 Year

**Characteristic**	**liraglutide for obesity**[1] (n=227)	**liraglutide for T2D**[1] (n=1,444)	**semaglutide for obesity** (n=377)	**semaglutide for T2D**[1] (n=1,341)
Persistence at 1 year,[2] *mean (SD)*				
Persistent	-5.6 (6.9)	-3.1 (6.1)	-12.9 (8.3)	-5.9 (7.3)
90-275 covered days	-3.4 (7.6)	-1.7 (6.5)	-5.2 (9.0)	-3.6 (6.5)
<90 covered days	-0.8 (7.2)	-1.2 (5.8)	-3.7 (8.0)	-2.0 (6.7)

N = 3389. Abbreviations: SD, standard deviation; T2D, type 2 diabetes. [1]Medication indication was categorized based on the brand name and presence of T2D. [2]Persistence with injectable semaglutide and liraglutide at 1 year was defined as a cumulative gap of <90 days within the first year after the initial prescription fill. We grouped non-persistent patients into those who had 90-275 medication coverage days within the first year and those with <90 covered days, respectively.

© 2024 Gasoyan H, et al. *JAMA Network Open*.

eTable 2. Cumulative Distribution of Categorical Weight Loss at 1 Year in the Overall Cohort

Categorical weight reduction	**liraglutide for obesity**[1] (n=227), No. (%)	**liraglutide for T2D**[1] (n=1,444), No. (%)	**semaglutide for obesity**[1] (n=377), No. (%)	**semaglutide for T2D**[1] (n=1,341), No. (%)
≥5%	84 (37.0%)	382 (26.5%)	209 (55.4%)	559 (41.7%)
≥10%	33 (14.5%)	134 (9.3%)	141 (37.4%)	223 (16.6%)
≥15%	12 (5.3%)	40 (2.8%)	80 (21.2%)	86 (6.4%)
≥20%	5 (2.2%)	17 (1.2%)	41 (10.9%)	43 (3.2%)

N = 3389. Abbreviations: T2D, type 2 diabetes. [1]Medication indication was categorized based on the brand name and presence of T2D.

© 2024 Gasoyan H, et al. *JAMA Network Open*.

eTable 3. Results of the Sensitivity Analysis, After Excluding Patients Who Switched Between the Medications of Interest From the Multivariable Model

Characteristic	Adjusted odds ratio (95% confidence interval) [1]	P value
GLP-1 RA agent		<0.001
liraglutide	reference	
semaglutide	2.11 (1.70-2.63)	
Indication[2]		<0.001
type 2 diabetes	reference	
obesity	2.46 (1.82-3.32)	
Medication dosage[3]		0.005
low	reference	
high	1.67 (1.16-2.40)	
Baseline BMI (1 unit increase)	1.02 (1.00-1.03)	0.01
Medication persistence at 1 year[4]		<0.001
<90 covered days	reference	
90-275 medication coverage days	1.51 (1.11-2.08)	
persistent	3.34 (2.50-4.51)	
Age (1 year increase)	1.01 (1.00-1.02)	0.23
Sex		
male	reference	<0.001
female	1.59 (1.29-1.97)	
Race/ethnicity		
While	reference	
Black	0.73 (0.54-0.96)	0.09
Hispanic	1.00 (0.66-1.48)	
Other[5]	1.35 (0.80-2.22)	
Not reported	1.71 (0.55-4.42)	
Charlson comorbidity index (1 unit increase)	0.98 (0.91-1.05)	0.50
Area deprivation index quartile[6]		
quartile 1: 1-25	reference	
quartile 2: 26-50	1.24 (0.88-1.76)	
quartile 3: 51 to 75	0.97 (0.69-1.38)	0.33
quartile 4: 76 to 100	1.19 (0.83-1.72)	
Unknown	0.97 (0.58-1.59)	
Primary payor		
Private	reference	
Medicare	1.35 (1.00-1.81)	
Medicaid	1.02 (0.71-1.43)	0.32
Self-Pay	0.46 (0.11-1.30)	
Other	1.20 (0.34-3.24)	
Unknown	1.04 (0.05-6.91)	
Receipt of other AOM before index date[7]		
None	reference	0.42
Prior use of other AOM	0.85 (0.56-1.26)	

© 2024 Gasoyan H, et al. *JAMA Network Open*.

N = 3306. Abbreviations: AOM, anti-obesity medication; BMI, body mass index; GLP-1 RA, glucagon-like peptide-1 receptor agonist. [1]Based on a multivariable logistic regression model with the following independent predictors in it: GLP-1 RA agent, medication indication, dosage, persistence with GLP-1 RAs at 1 year, age, sex, race/ethnicity, primary payor type, ADI quartile, baseline BMI, age-adjusted Charlson comorbidity index, and previous use of other anti-obesity medication. [2]Medication indication was categorized based on the brand name and presence of T2D. [3]Medication maintenance dosage was dichotomized with semaglutide 1.7 mg, 2.0 mg, 2.4 mg, and liraglutide 3 mg classified as "high dose" and all other dosages classified as "low dose." [4]Persistence with injectable semaglutide and liraglutide at 1 year was defined as a cumulative gap of <90 days within the first year after the initial prescription fill. We grouped non-persistent patients into those who had 90-275 medication coverage days within the first year and those with <90 covered days, respectively. [5]Other race/ethnicity included American Indian or Alaska Native, Asian, Multiracial, Pacific Islander, and other. [6]ADI quartiles were structured by ranking the ADI from low to high nationally, where an ADI with a ranking of 1 indicates the lowest level of disadvantage. [7]Includes receipt of phentermine-topiramate, naltrexone-bupropion, or orlistat before the initial fill for liraglutide or semaglutide.

© 2024 Gasoyan H, et al. *JAMA Network Open*.

eFigure. Cumulative Distribution of Categorical Weight Reduction Percentage at 1 Year Among Patients Who Persisted With the Medications of Interest

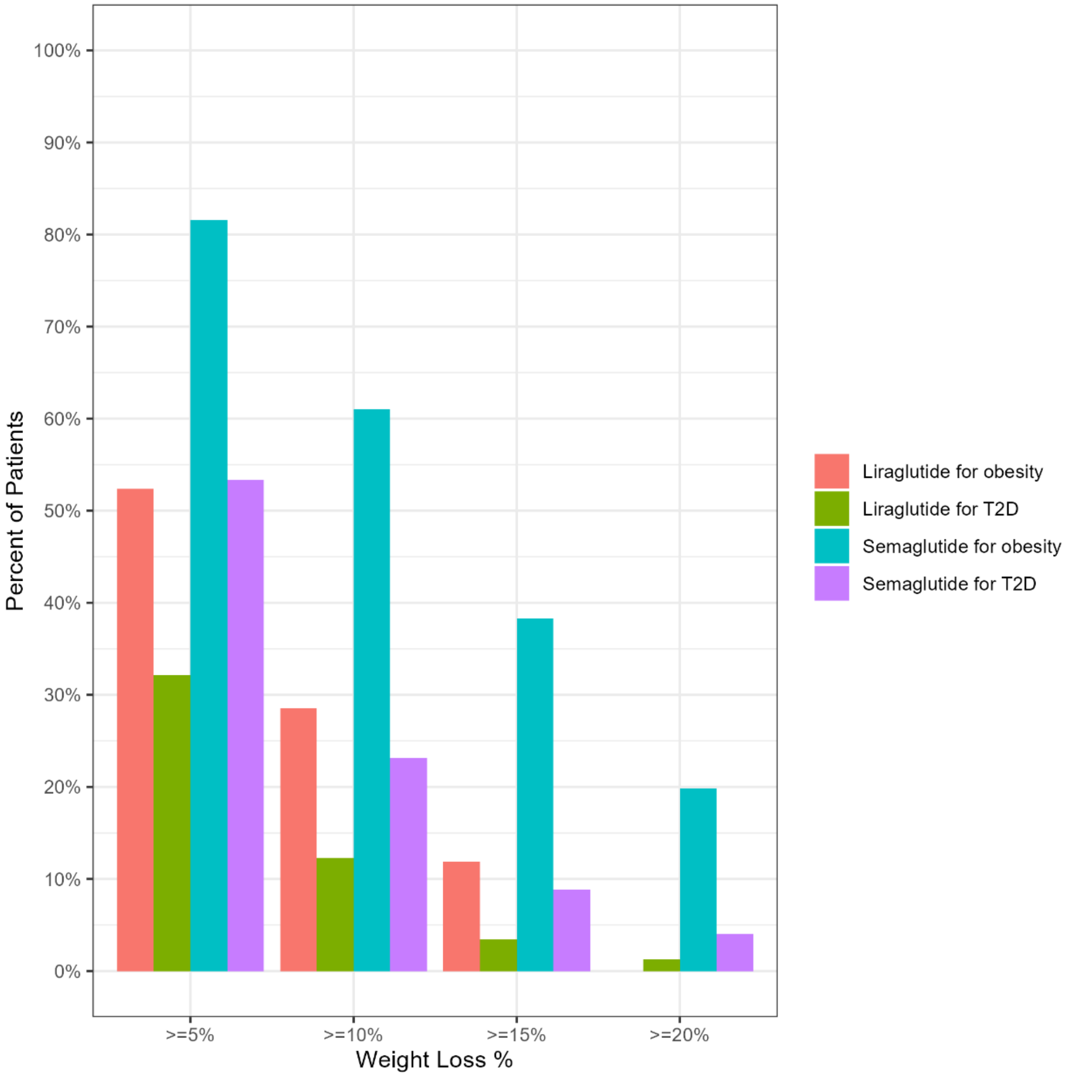

Categorical weight reduction at year 1	**liraglutide for obesity**[1] (n=42), No. (%)	**liraglutide for T2D**[1] (n=553), No. (%)	**semaglutide for obesity**[1] (n=141), No. (%)	**semaglutide for T2D**[1] (n=645), No. (%)
≥5%	22 (52.4%)	178 (32.2%)	115 (81.6%)	344 (53.3%)
≥10%	12 (28.6%)	68 (12.3%)	86 (61.0%)	149 (23.1%)
≥15%	5 (11.9%)	19 (3.4%)	54 (38.3%)	57 (8.8%)
≥20%	0 (0%)	7 (1.3%)	28 (19.9%)	26 (4.0%)

N = 1381. Abbreviations: T2D, type 2 diabetes. [1]Medication indication was categorized based on the brand name and presence of T2D. Persistence with injectable semaglutide and liraglutide at 1 year was defined as a cumulative gap of <90 days within the first year after the initial prescription fill.

© 2024 Gasoyan H, et al. *JAMA Network Open*.
