# Supplementary material for: One-Year Weight Reduction With Semaglutide or Liraglutide in Clinical Practice
Source: JAMA Netw Open. 2024 Sep 13;7(9):e2433326. doi: 10.1001/jamanetworkopen.2024.33326 (PMC11400221; doi:10.1001/jamanetworkopen.2024.33326)
Supplement: Supplement 2. — Data Sharing Statement [file jamanetwopen-e2433326-s002.pdf]

Data Sharing Statement

Gasoyan. One-Year Weight Reduction With Semaglutide or Liraglutide in Clinical Practice. *JAMA Netw Open*. Published September 13, 2024. doi:10.1001/jamanetworkopen.2024.33326

Data
Data available: No

Additional Information
Explanation for why data not available: The datasets generated during and/or analyzed during the current study are not publicly available to protect patient confidentiality.
